# Influence of the hole geometry on the flow distribution in ventricular catheters for hydrocephalus

**DOI:** 10.1186/s12938-016-0182-1

**Published:** 2016-07-15

**Authors:** Ángel Giménez, Marcelo Galarza, Olga Pellicer, José Valero, José M. Amigó

**Affiliations:** 1 Operations Research Center, University Miguel Hernández de Elche, Avda. Universidad s/n, 03202 Elche (Alicante), Spain; 2 Regional Department of Neurosurgery, Virgen de la Arrixaca University Hospital, 30120 El Palmar, Murcia, Spain; 3 Department of Health Psychology, University Miguel Hernández de Elche, Avda. Universidad s/n, 03202 Elche (Alicante), Spain

**Keywords:** Hydrocephalus, Ventricular catheter, Computational fluid dynamics, Hole geometry, Shear stress, Flow rate

## Abstract

**Background:**

Hydrocephalus is a medical condition consisting of an abnormal accumulation of cerebrospinal fluid within the brain. A catheter is inserted in one of the brain ventricles and then connected to an external valve to drain the excess of cerebrospinal fluid. The main drawback of this technique is that, over time, the ventricular catheter ends up getting blocked by the cells and macromolecules present in the cerebrospinal fluid. A crucial factor influencing this obstruction is a non-uniform flow pattern through the catheter, since it facilitates adhesion of suspended particles to the walls. In this paper we focus on the effects that tilted holes as well as conical holes have on the flow distribution and shear stress.

**Methods:**

We have carried out 3D computational simulations to study the effect of the hole geometry on the cerebrospinal fluid flow through ventricular catheters. All the simulations were done with the OpenFOAM® toolbox. In particular, three different groups of models were investigated by varying (i) the tilt angles of the holes, (ii) the inner and outer diameters of the holes, and (iii) the distances between the so-called hole segments.

**Results:**

The replacement of cylindrical holes by conical holes was found to have a strong influence on the flow distribution and to lower slightly the shear stress. Tilted holes did not involve flow distribution changes when the hole segments are sufficiently separated, but the mean shear stress was certainly reduced.

**Conclusions:**

The authors present new results about the behavior of the fluid flow through ventricular catheters. These results complete earlier work on this topic by adding the influence of the hole geometry. The overall objective pursued by this research is to provide guidelines to improve existing commercially available ventricular catheters.

## Background

Hydrocephalus is a medical condition characterized by an excessive accumulation of cerebrospinal fluid (CSF) in the ventricles of the brain. The ventricles are four cavities inside the brain, and the CSF is a colorless liquid surrounding the brain and the spinal cord. The most common treatment for hydrocephalus is the insertion of a cerebrospinal fluid shunt system. The shunt system comprises three parts: distal catheter, shunt valve and ventricular catheter. The standard ventricular catheter (VC) is a flexible tubing with a number of holes placed symmetrically around several transversal sections or “drainage segments” which is inserted in one of the ventricles to extract the excess fluid. The valve regulates the pressure and so the outlet flow.

Between 50 and 80 % of all shunt malfunctions are caused by obstruction of the VC. The most common cause of VC obstruction is the adhesion of cells and macromolecules to the walls of the catheter which, in turn, is due to a variety of factors, including wrong placement of the catheter and the material used. But some of the most important factors are inherent to the geometric configuration of the catheter since it determines the flow characteristics and the wall shear stress at the holes (see [1–9]).

In [10–13], the authors studied the effect of some parameters variation on the flow distribution by using three-dimensional computational fluid dynamics (CFD). These parameters included the hole size but not its geometry (cylindrical vs conical holes) nor its angular position (perpendicular vs tilted) with respect to the wall. In this paper we fill this gap and show that both geometrical features are relevant as well for the flow characteristics.

## Methods

### CFD software

A numerical model consists of three basic steps regarding its performance: pre-processing, solving process, and post-processing. In the pre-processing the computational domain and the meshing of the model are built, and the initial and boundary conditions are fixed. Next, a suitable numerical scheme is implemented to solve the governing equations of the model. Lastly, in the post-processing, a correct analysis and visualization of the data are required to ensure a proper discussion of the results.

It is therefore customary in CFD to use numerous software tools to carry out a numerical experiment. The core of our simulations was OpenFOAM®, which is the acronym of *Open Source Field Operation and Manipulation*. OpenFOAM® is an open-source CFD software based on C++ that contains a toolbox for tailored numerical solvers. The algorithm implemented in OpenFOAM® uses the Finite Volume Method on unstructured meshes (see [14, 15]). OpenFOAM® includes pre-processing and post-processing capabilities such as snappyHexMesh and ParaFoam for meshing and visualization, respectively. We also used other open-source software that provide pre-processing and post-processing tools for CFD and numerical simulations such as Salome and ParaView. Salome (version 7.3.0) was mainly used to build the geometry of the different models, and ParaView (version 4.1.0) to display some of the images.

### Flow domain

**Fig. 1:**
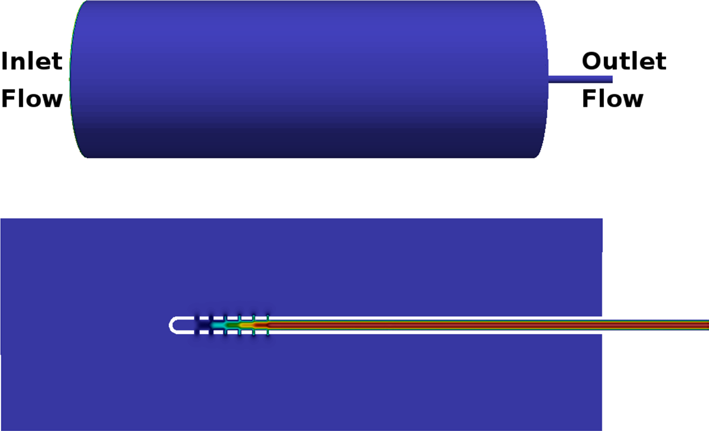
Flow domain.* Upper panel*: The three-dimensional computational domain in all numerical simulations.* Lower panel*: A two-dimensional slice of the computational domain

The structural and geometric complexity of the ventricles prevents from implementing an accurate computational model. On the other hand, we are only interested in the CSF flow pattern in the proximity of the VC. For this purpose it suffices that the flow domain of all our models is a catheter inserted into a cylinder through one of its bases, as in Fig. 1. To comply approximately with the standard ventricle volume, the cylinder is 85 mm long and its diameter is 30 mm. The inner diameter and outer diameter of the catheter are 1.5 and 2.5 mm, respectively, and the part of the catheter inside the cylinder has a length of 60 mm. The assumption that both the cylinder and the catheter are rigid and straight is a natural simplification that implies no significant differences in the results.

The computational domain was created bottom-up with Salome and converted to a stereolithography to be linked to the snappyHexMesh utility for the generation of the mesh. An unstructured grid based on hexahedral meshes was generated. Mesh cells are the atomic elements on which the physics of the flow is solved, and hexahedral meshes give more accurate solutions, especially when the grid lines are aligned with the flow. Refined meshes around the holes were created to capture all relevant flow features under study.

### Governing equations

Our model is governed by the incompressible Navier–Stokes equations given by:

1 $$\begin{aligned} \frac{\partial \mathbf {u}}{\partial t}+\mathbf {u}\cdot \nabla \mathbf {u}-\nu \Delta \mathbf {u}+\nabla p&= \mathbf {0} \end{aligned}$$

2 $$\begin{aligned} \nabla \cdot \mathbf {u}&= 0 \end{aligned}$$

where $\mathbf {u}$ and *p* stand for the velocity field and the pressure, respectively, $\nu$ denotes the kinematic viscosity of the CSF ($\nu =7.5\times 10^{-7}$ m$^{2}$/s), and *t* the time. Equation (1) is the general Navier–Stokes equation. Fluids described by this equation are called simple (or newtonian) because their stress tensor has a mathematically simple structure. The Navier-Stokes equation is thought to describe simple fluids even in the turbulent regime. Equation (2) is the incompressibility constraint, which entails in particular that the inlet flow is equal to the outlet flow. The most important simple, incompressible fluid is, of course, water. The cerebrospinal fluid behaves to a high degree of accuracy as a simple fluid, what justifies the use of Eqs. 1 and 2 in this case. The reynolds number of the fluid increases from 0 (inital time) to about 20 (stationary regime). Therefore, the fluid is laminar all the time since turbulence sets in typically at reynolds numbers larger than 5000.

We integrated numerically the coupled system (1) and (2) by means of the icoFoam solver, which is based on the so-called PISO algorithm and implemented in OpenFOAM®. The icoFoam code is inherently transient, requiring an initial condition and boundary conditions. The flow was computed till stationarity set in. The convergence of the solver was highly successful, except in a few cases which were solved by refining the mesh to avoid skewed cells.

### Initial and boundary conditions

All non-stationary CFD problems require initial and boundary conditions, these being necessary to specify the initial values of all the flow variables at all points in the computational domain. The initial and boundary conditions described in the paragraph below are the same as in [2, 5, 7, 8].

At the inlet boundary (the left cylinder base in Fig. 1), the velocity field was adjusted to achieve a constant inflow of 100 cm^3^/day, and the pressure was taken as zero gradient. At the outlet boundary (the intersection of the VC lumen with the right cylinder base) the pressure was fixed to 15 cm H_2_O (=1471 Pa) and a zero gradient condition was set on the velocity. The conditions at the wall boundaries were nonslip for the the velocity and zero gradient for the pressure. On the rest of the computational domain the initial velocity was taken to be zero and the pressure 15 cm H_2_O.

### Description of the models

**Fig. 2:**
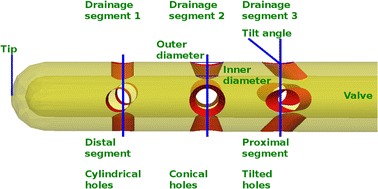
Elements of a catheter. Schematic representation of the component parts of the catheter used throughout the paper

Before going into the models we shall briefly explain the terminology used later on for the elements of the catheter. Figure 2 displays a VC with three drainage segments which will be always numbered from the tip to the valve (left-to-right). Drainage segments are also called hole segments or just segments. We refer to the closest segment to the tip of the catheter as the distal segment. Similarly, the proximal segment stands for the closest segment to the valve. Three types of holes can be distinguished in Fig. 2: cylindrical (segment 1), conical (segment 2) and tilted (segment 3), where in the latter case the holes could in turn be cylindrical or conical. In a conical hole, the largest (resp. smallest) diameter is the outer (resp. inner) diameter with respect to the VC lumen, and its surface area will be called the outer (resp. inner) area of the hole. We will refer to the sum of the outer and inner areas of all holes located on the same drainage segment as the drainage outer area and the drainage inner area of that segment, respectively.

Although relative rotations of the drainage segments do not change the flow rate distribution per segment [11, 13], it is convenient to rotate contiguous segments to achieve a higher mechanical stability, especially when they are very close. For a better visualization of the images though, we did not always follow this recommendation in our models.

For studying the effect of different hole shapes on the flow, we divided the models into the four groups described below. All models from 1 to 10 have the following common feature: 3 drainage segments with 4 holes with an inner diameter of 0.5 mm each. Owing to the number of parameters involved (tilt angle, outer cross section and inter-segment distance) and time-demanding modelization and computation, the number of models studied was limited but sufficient for our purposes, namely, to derive qualitative principles for VC design.

**Table 1:** Geometrical parameters of the VCs in Group I, II and III

	Outer diameter	Tilt angle	Distance to the tip
*Group I*			
Model 1	(0.5, 0.5, 0.5)	(0.0, 0.0, 45.0)	(4.0, 10.0, 16.0)
Model 2	(0.5, 0.5, 0.5)	(0.0, 45.0, 0.0)	(4.0, 10.0, 16.0)
Model 3	(0.5, 0.5, 0.5)	(45.0, 0.0, 0.0)	(4.0, 10.0, 16.0)
*Group II*			
Model 4	(0.5, 0.5, 1.0)	(0.0, 0.0, 0.0)	(4.0, 10.0, 16.0)
Model 5	(0.5, 1.0, 0.5)	(0.0, 0.0, 0.0)	(4.0, 10.0, 16.0)
Model 6	(1.0, 0.5, 0.5)	(0.0, 0.0, 0.0)	(4.0, 10.0, 16.0)
*Group III*			
Model 7	(1.3, 1.1, 0.5)	(0.0, 0.0, 0.0)	(4.0, 6.0, 16.0)
Model 8	(1.3, 0.94, 0.5)	(0.0, 0.0, 0.0)	(4.0, 8.0, 16.0)
Model 9	(1.3, 0.8, 0.5)	(0.0, 0.0, 0.0)	(4.0, 10.0, 16.0)
Model 10	(1.3, 0.68, 0.5)	(0.0, 0.0, 0.0)	(4.0, 12.0, 16.0)

Each entry in a bracket displays the corresponding measure in the following order: (distal segment, middle segment, proximal segment). Lengths are given in millimeters and angles in degrees. All the parameters are illustrated in Fig. 2

Group I consists of three models (1, 2 and 3 described in Table 1), each model having one drainage segment with all its holes tilted 45 degrees from the vertical (see Fig. 2). The segments are located at 4/10/16 mm from the tip.

Group II consists of three models (4, 5 and 6 described in Table 1) each model having one drainage segment with all its holes being conical. The drainage segments are located as in Group I.

Group III consists of four models, each model differing from the other in that the middle segment is shifted. But before dealing with the issue of how to choose inter-segment distances, we shall discuss how to fix the diameters of the holes to get a more even flow distribution. In view of the results obtained with Group II, it seems appropriate to consider catheters in which the drainage outer area decreases from the distal segment to the proximal segment. Numerical simulations show that, indeed, it is suitable to take exponentially decreasing drainage outer areas. This being the case, we are going to adopt the following criterion for selecting drainage outer areas.

Suppose that we want to design a catheter with *N* segments numbered distal-to-proximal by *i*, $1\le i \le N$, with a certain number of holes each one. Let $A_i$ be the drainage outer area of the segment *i*, and let $d_i$ be the distance from the tip of the catheter to the center of the segment *i*. We first set the areas $A_1$ and $A_N$ ($A_1\ge A_N$) of the distal and proximal segments, respectively, as well as the positions of all segments, i.e. $d_{1}$, $d_{2},\ldots , d_{N}$. The outer areas $A_i$ of the intermediate segments, $1<i<N$, are then calculated by the mathematical formula

3 $$\begin{aligned} A_{i}=A_{1} e^{\lambda (d_i-d_1)};\quad \lambda =\frac{1}{d_{N}-d_{1}}\ln \left( \frac{A_N}{A_1}\right) . \end{aligned}$$

Observe that this formula is suitable provided that $A_{1}\ge A_{N}$ (or equivalently, $\lambda \le 0$), i.e., when the drainage outer area of the distal segment is equal or greater than the drainage outer area of the proximal segment. Formula (3) was also applied in [13] to compute drainage inner areas instead of drainage outer areas. We show below that this novel application of formula (3) has similar homogenizing effects on the flow rate per segment, but with the advantage of not having to reduce the inner diameter of the holes, i.e., the hole cross section. Indeed, the smaller the hole cross section, the more prone the hole is to get obstructed. As a final remark, the formula (3) was not derived from first principles, but it is rather an empirical ansatz.

Bearing in mind this general criterion, we have considered four configurations in Group III (models 7, 8, 9 and 10 described in Table 1) to analyze numerically how different inter-segment distances affect the flow rate distribution.

**Table 2:** Geometrical parameters of the VCs in Group IV

	Outer diameter	Tilt angle	Distance to the tip
Model 11	(0.5, 0.5, 0.5, 0.5, 0.5, 0.5)	(0.0, 0.0, 0.0, 0.0, 0.0, 0.0)	(4.0, 5.5, 7.0, 8.5, 10.0, 11.5)
Model 12	(0.8, 0.66, 0.66, 0.54, 0.62, 0.5)	(0.0, 0.0, 0.0, 0.0, 0.0, 0.0)	(4.0, 5.5, 7.0, 8.5, 10.0, 11.5)
Model 13	(0.8, 0.66, 0.66, 0.54, 0.62, 0.5)	(0.0, 15.0, 15.0, 15.0, 15.0, 45.0)	(4.0, 5.5, 7.0, 8.5, 10.0, 11.5)

Each entry in a bracket displays the corresponding measure in the order from the distal segment to the proximal segment. Lengths are given in millimeters and angles in degrees. All the parameters are illustrated in Fig. 2

Group IV consists of three models (11, 12 and 13 described in Table 2) designed to illustrate the benefits of combining conical holes with different tilt angles and formula (3) to select the outer areas of the drainage segments. The three models 11, 12 and 13 have six drainage segments with 6, 6, 4, 4, 2 and 2 holes, each with an inner diameter of 0.5 mm. In model 11, all the holes are cylindrical. In model 12, the geometric configuration is the same as in model 11, but the holes are conical instead, and the outer area follows the formula (3). In model 13, the geometric configuration is the same as in model 12, but some holes were tilted.

## Results

**Fig. 3:**
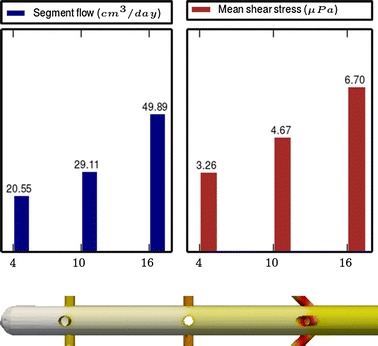
**Results of Model 1**

**Fig. 4:**
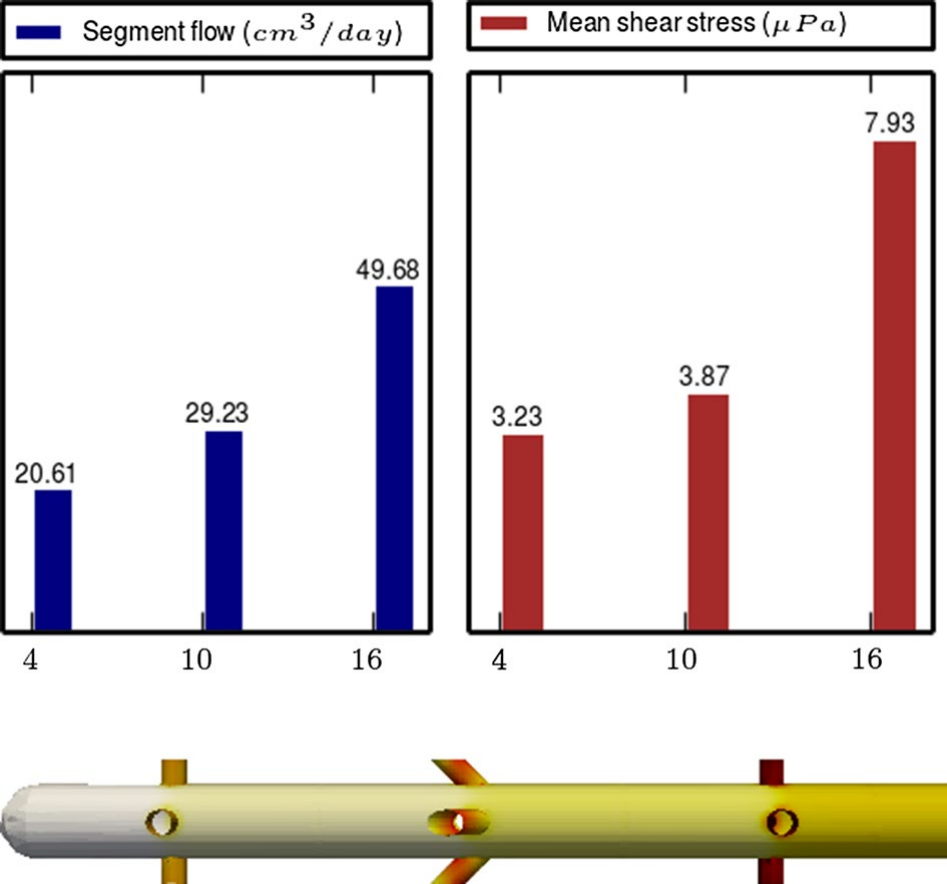
**Results of Model 2**

**Fig. 5:**
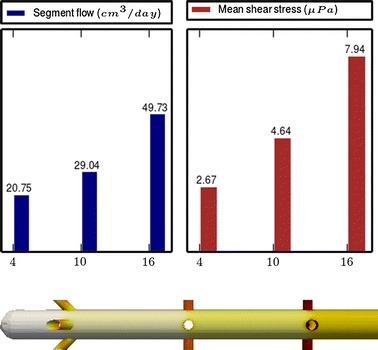
**Results of Model 3**

**Fig. 6:**
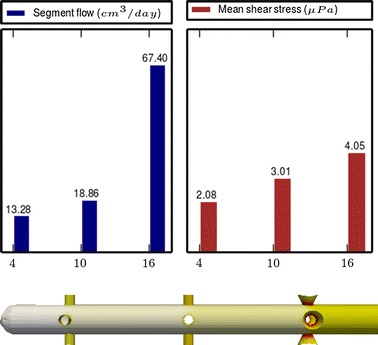
**Results of Model 4**

**Fig. 7:**
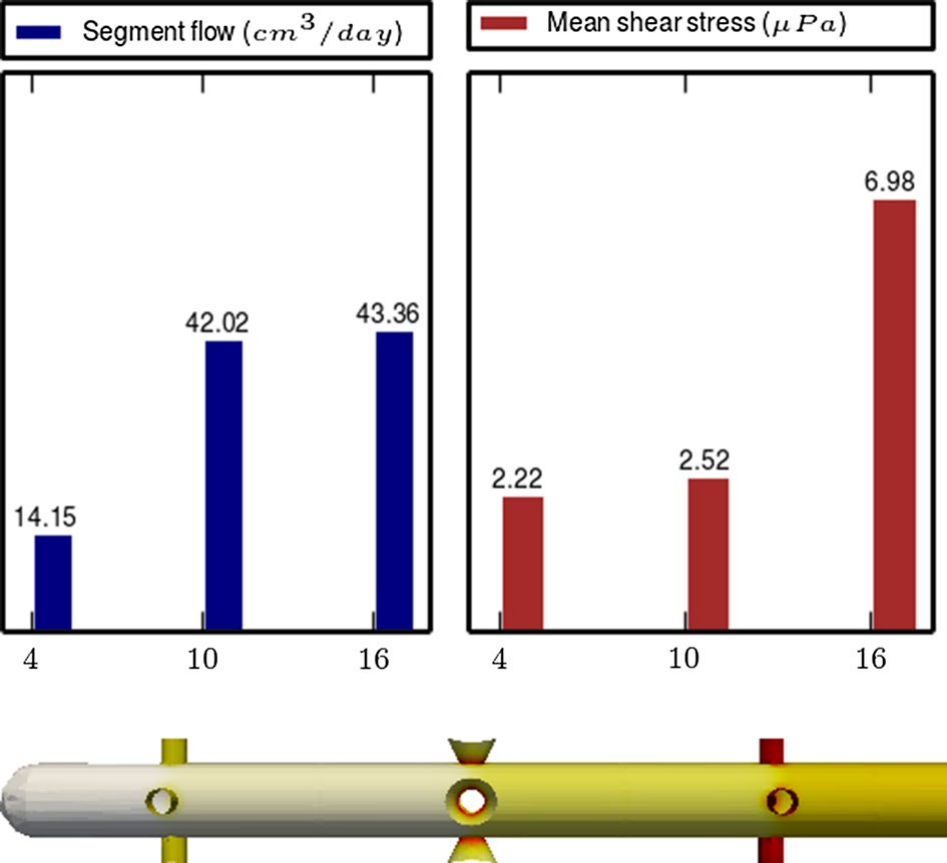
**Results of Model 5**

**Fig. 8:**
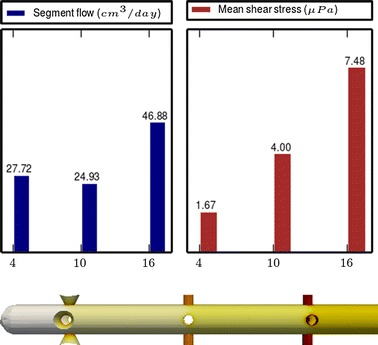
**Results of Model 6**

**Fig. 9:**
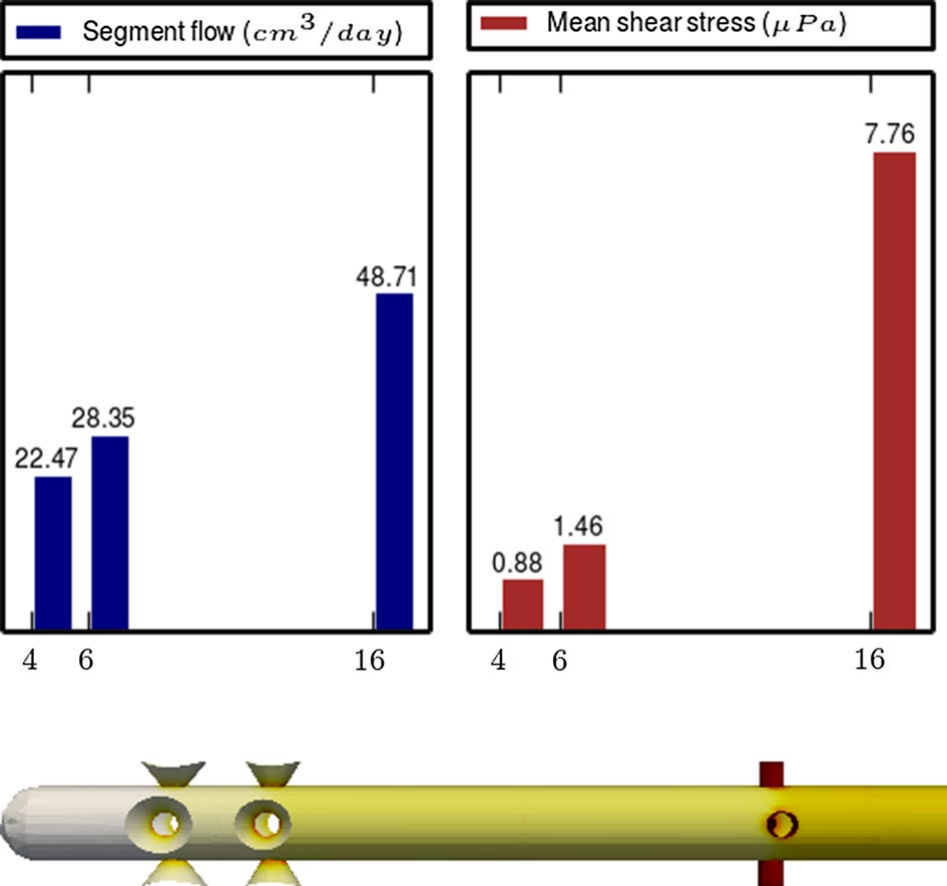
**Results of Model 7**

**Fig. 10:**
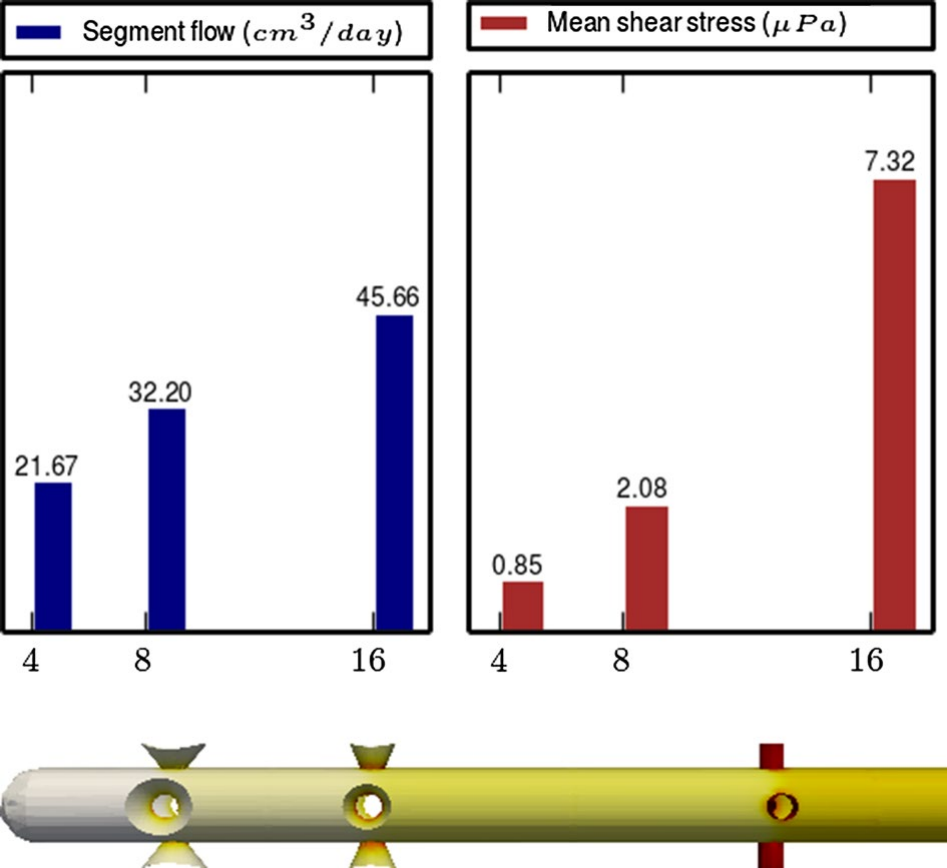
**Results of Model 8**

**Fig. 11:**
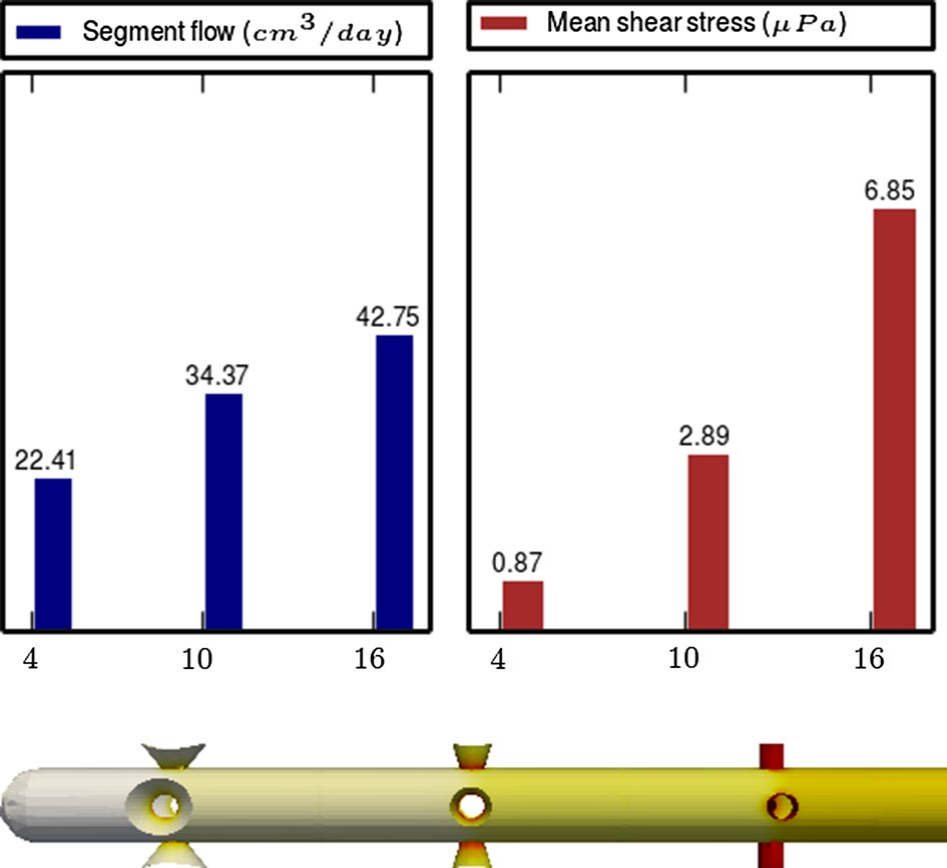
**Results of Model 9**

**Fig. 12:**
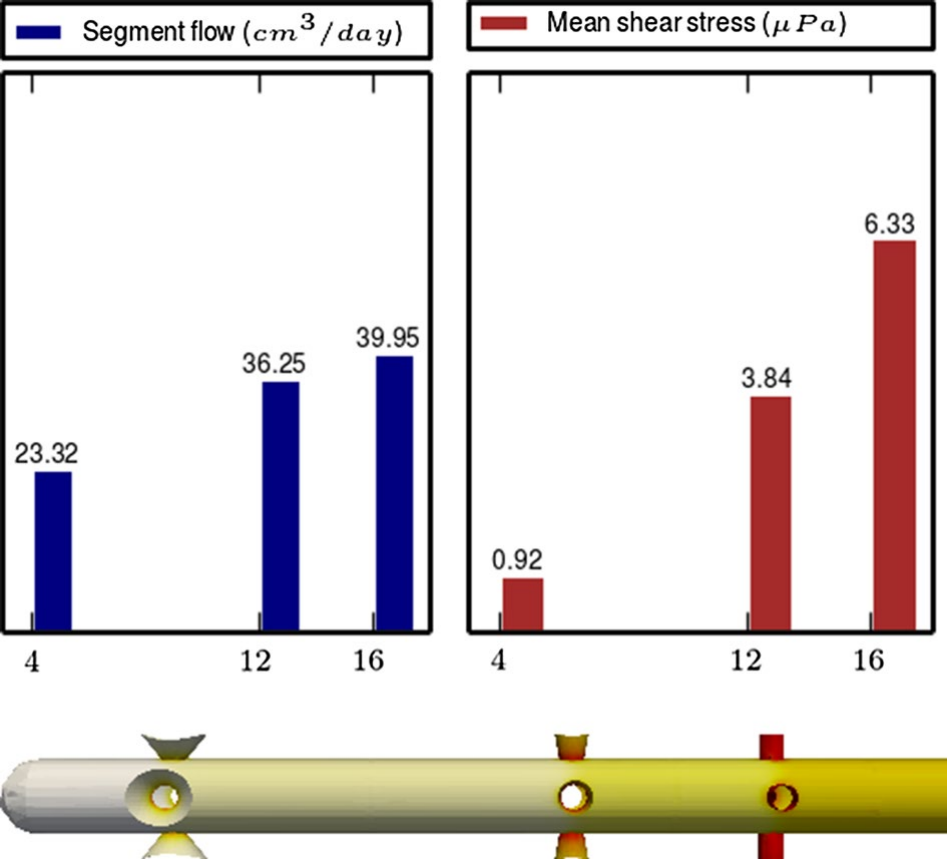
**Results of Model 10**

**Fig. 13:**
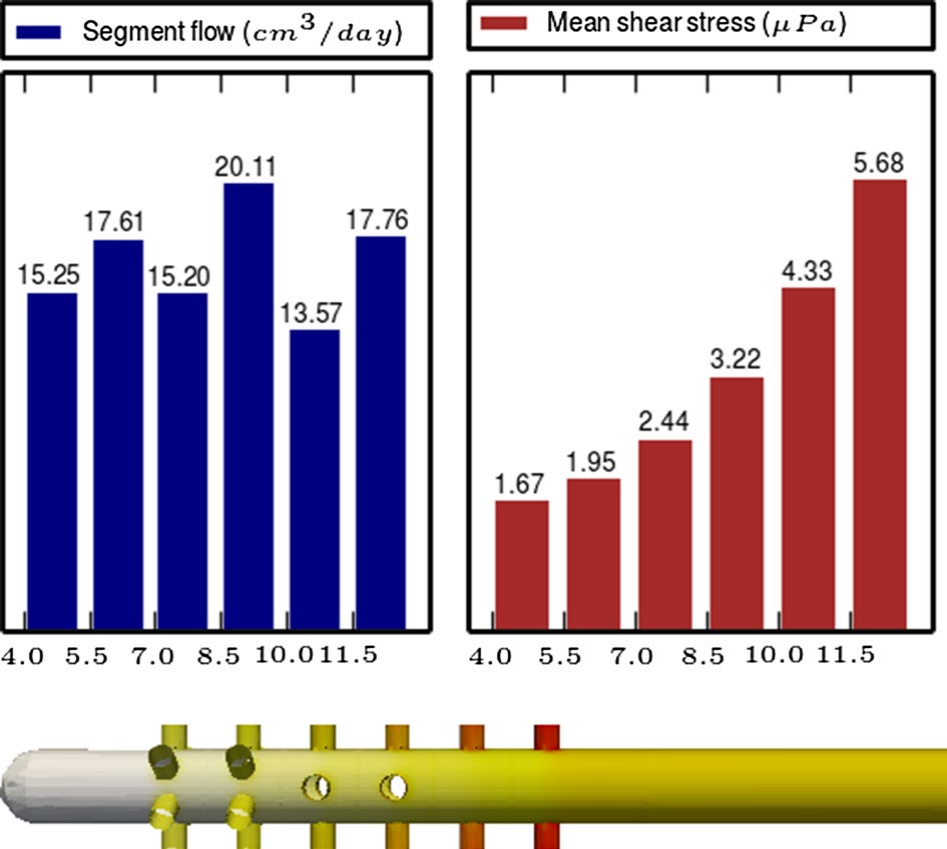
**Results of Model 11**

**Fig. 14:**
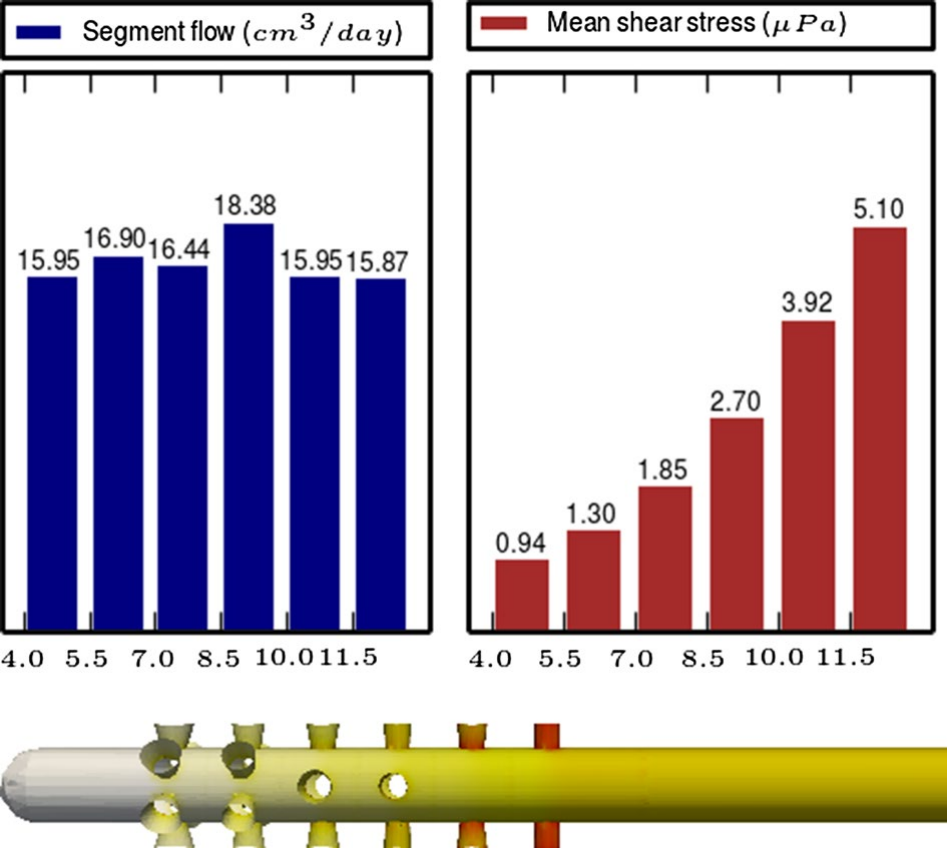
**Results of Model 12**

**Fig. 15:**
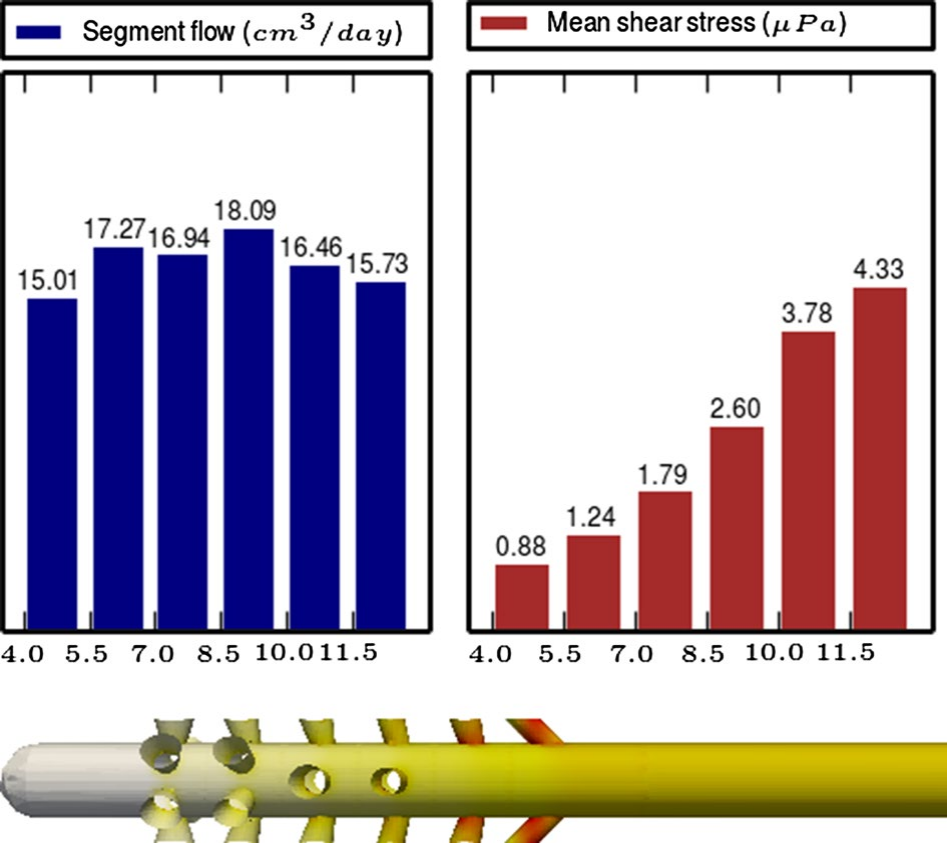
**Results of Model 13**

The main scope of this study was to analyze the distribution of the flow rate and mean shear stress per segment for the different models. All the results are shown in Figs. 3, 4, 5, 6, 7, 8, 9, 10, 11, 12, 13, 14 and 15 each one having the same description. In the upper part there are two bar graphs depicting the flow rate per segment (left), and the mean shear stress on the walls of all holes of the same segment (right). In the lower part the shear stress distribution on the inner wall and hole of the catheter is color coded, the darker the color, the higher the shear stress. In all models, the flow is given in cm^3^/day and the shear stress in $\mu$Pa. Since the total inflow (and so the total outflow) is 100 cm^3^/day, the results for the segments flow can also be considered as flow percentages. Also in all models, the inner diameter of the holes is 0.5 mm.

### Group I: Influence of the hole tilt

Results of the Group I are displayed in Figs. 3, 5 and 6. They show the effect of segments with tilted holes. We may assert that no significant differences in the segment flow rates emerged between the three models tested, although we should note that this holds because the distance between the segments is large (4 mm in this case). In fact, according to [13], the closer the segments are, the more sensitive is the flow rate with respect to the tilt angle. On the contrary, important differences were found for the mean shear stress. In particular, when tilted holes are included in any segment, the mean shear stress is reduced approximately 20 %.

The numerical simulations also disclosed (not shown here) that the higher the tilt angle, the lower the mean shear stress. However, tilt angles higher than 45° might cause mechanical stability problems.

### Group II: Influence of conical holes

Figures 7, 8 and 9, show the effect of segments with conical holes (models 4, 5 and 6, respectively). It is worth observing that conical holes boost the “sink effect” by increasing the flow passing through them. In our numerical experiments (not all shown here), the flow growth rates obtained when cylindrical holes are replaced by conical holes were approximately the following: 35 % at the distal segment (Model 4), 45 % at the middle segment (Model 5) and 35 % at the proximal segment (Model 6). Moreover, at the same time that the segment flow rate increases with conical holes, the mean shear stress per segment is also slightly reduced. Thus, if the conical holes are properly placed in the catheter, they will increase the segment flow rate and decrease the mean shear stress.

### Group III. Influence of conical holes with varying inter-segment distance

The most significant conclusion from this group is that constant inter-segment distances is not the best arrangement. Indeed, the results of the numerical experiments shown in Figs. 9, 10, 11 and 12 clearly indicate that, in a three-segment catheter configuration, the distribution of the segment flow rates improves (i.e., gets more uniform) as the middle segment shifts from left to right. This suggests that a segment distribution with diminishing distances from the distal position to the proximal position is a better choice than keeping constant the inter-segment distances.

### Group IV. Application example

The numerical experiments in Group IV show the homogenizing effect on the segment flow rate distribution when we move from cylindrical holes to conical holes according to the formula (3). We start from Model 11 (see Fig. 13), whose design with cylindrical holes produces a non-uniform segment flow rate distribution. As a first step, in Model 12 (see Fig. 14) the cylindrical holes are replaced by conical ones following the formula (3), obtaining this way a more uniform distribution and a slight reduction of the shear stress. As a second step, in Model 13 (see Fig. 15) some holes are tilted to achieve further improvements in the flow distribution and shear stress because of the effects studied with the models of Group I and III.

## Discussion

In this study we evaluated the fluid dynamics of several VC prototypes to analyze flow factors related specifically to the hole geometry. These results will contribute to better understand the fluid-mechanical causes of VC malfunction in hydrocephalus, thus possibly helping to prolong the VC lifetime. Indeed, hydrocephalus is being treated with a shunt system which drains CSF from the cerebral ventricles, but its significant failure rate has caused engineers and neurosurgeons to query its design. The most common failure site is the ventricular catheter due to occlusion by debridement, cells, and brain tissue [1, 4, 5, 7, 9]. According to the literature ([1–9]), the flow distribution and the shear stress play an important role in such an adhesion of particles to walls. In turn, the flow distribution and the shear stress are determined by the geometric configuration of the catheter. Let us remind that the shear stress is a measure of the force of friction from a fluid acting on a body in the path of the fluid. The experimental evidence shows that the closest holes to the valve in a VC (i.e., belonging to the most proximal segments) are the primary sites of blockage. Of course, obstruction in the proximal perforated area in vivo may be caused also by other reasons, including holes being positioned outside the ventricle. Interestingly, manufacturers have barely altered the number of holes and their placement over the last 60 years [5, 7]. There are no overtly accessible records indicative of why the number of holes, the size of each hole, and the distance between segments were so chosen [7]. Historically, the general consensus has been that the number and size of the holes were chosen to facilitate an adequate flow from the ventricular space, but recent studies suggest that perhaps fewer holes are required for this [6, 9]. The first study of VCs that varied the drainage hole size was done by Lin et al. [1]. They concluded that a gradual decrease in hole diameter from the catheter tip would modify the mass flow rate distribution and would hypothetically reduce the probability of occlusion. Yet, this latter key conclusion was not tested. Notably, the shear stress through a hole depends on its size, a factor known to control cell adhesion [16].

The only study today that have specifically addressed the influence of the hole size on cell adhesion was done by Harris et al. [7]. As the VC of the shunt system is made of poly(dimethyl)siloxane (PDMS), they designed several PDMS samples, designed to mimic the current clinical catheter, with holes that varied in diameter. The number of holes was so chosen that the bulk flow rate and total hole surface area were equal across all samples. The holes were cylindrical or conical holes according to the punching fabrication method used. Analysis of the data using linear regression suggested that the relationship between one cell type (astrocyte) adhesion and hole size was more linear than the relationship between other cell type (macrophage) adhesion and hole size. Their results implied a dependency on how the holes were oriented in the flow system, and suggested that the flow distribution is not the only factor in adhesion. Another factor that may influence adhesion is gravity. They concluded that the cell type adhesion has a strong dependency on the hole size (and most probably on the shear stress as well), and therefore these relationships should be considered in future studies. However, in their study, the shear stress was calculated assuming that the volumetric flow rate through each hole and the hole diameter remained constant. Furthermore, they proposed to interpret the shear stress values only as approximations, because the volumetric flow rate per hole was not measured in their study and, moreover, it probably varies with the distance to the catheter tip, as suggested by Lin et al. [1]. As we used mathematical modeling and numerical simulation, our values are more accurate in that point.

Our models are based on “clean water”, so it might differ slightly from reality since the CSF contains cells and macromolecules. It is important to stress that our main purpose is not so much the search of precise data results, but rather the discussion of how the flow distribution and shear stress vary with the variation of the hole diameters and tilt angles of the holes. As said above, the flow distribution is the key factor in VC obstruction.

Pursuant to article [13], the basic principles of the segment flow distribution behavior can be summarized as follow:

- Relative rotations of the drainage segments around the axis of the catheter and rigid translations of all the segments along the catheter do not change the flow distribution.
- The flow distribution pattern depends on the inter-segment distances in a sensitive way. In particular, one can transfer fluid mass from right to left just by scaling down the inter-segment distances (and viceversa).
- A lower number of segments as well as a lower number of holes per segment favors a more uniform flow distribution pattern, but the mean shear stress increases.

In view of the results in this paper, to the above principles we should add the following:

- A decreasing distribution of outer diameters of holes (while keeping the inner diameters fixed) also causes transfer of fluid mass from the proximal perforated area to the distal one.
- The flow distribution is not much affected by the hole tilt when the distance between segments is large, however it has a positive effect on the mean shear stress distribution. On the contrary, tilted holes can have a considerable influence when the drainage segments are close enough.

## Conclusions

Current commercially available VCs are very often designed with no other guideline than geometrical symmetry. But the difficulties caused by their short mean lifetime require a more rigorous approach. In this regard, further work is needed to design new prototypes with (i) an even (or a decreasing) segment flow distribution pattern, (ii) holes as large as possible, (iii) shear stress as low as possible, and (iv) technical simplicity of manufacturing. The Rivulet model [17] was designed to attain a more uniform flow distribution than previous designs, but it also has the disadvantage of having too small hole diameters in the most proximal segments. The present work suggests an alternative solution by combining the use of conical holes (fixing the inner diameter and varying the outer diameter) and tilted holes. It is noteworthy that technically simple changes, which might be overlooked at first sight, become relevant to achieve better designs.
